# Advances in immunotherapy for renal cell carcinoma: a comprehensive review

**DOI:** 10.3389/fimmu.2026.1715874

**Published:** 2026-04-20

**Authors:** Emad Rajih, Abdulaziz Bakhsh, Walaa M. Borhan, Saeed Awad M. Alqahtani

**Affiliations:** 1 General and Specialized Surgery Department, Taibah University College of Medicine, Medina, Saudi Arabia; 2 Basic Medical Sciences Department, Taibah University College of Medicine, Medina, Saudi Arabia

**Keywords:** biomarkers, immune checkpoint inhibitors, renal cell carcinoma, resistance mechanisms, tumor microenvironment, tyrosine kinase inhibitors

## Abstract

This review examines the evolving first-line immunotherapy landscape in metastatic renal cell carcinoma (mRCC), with emphasis on the comparative clinical logic of dual immune checkpoint blockade (ICI-ICI) and immune checkpoint inhibitor-tyrosine kinase inhibitor combinations (ICI-TKI), the persistent efficacy-effectiveness gap, biomarker development, and translational resistance biology. Pivotal phase III trials have established superior survival for contemporary ICI-based regimens over sunitinib; however, cross-trial heterogeneity, differences in IMDC risk distribution, varying toxicity profiles, and selection of fitter trial populations complicate simple regimen ranking. Real-world studies confirm that outcomes in routine practice are frequently less favorable than those reported in registration studies, but these differences are partly explained by confounding related to performance status, comorbidity burden, access to care, toxicity management, and treatment sequencing. Renal cell carcinoma remains a biomarker-challenged disease in which PD-L1 and tumor mutational burden have limited predictive value, while PBRM1 status, VHL-driven pseudohypoxia, and spatial immune architecture are biologically informative but not yet clinically validated as stand-alone selection tools. Resistance arises through tumor-intrinsic metabolic reprogramming, impaired antigen presentation, compensatory checkpoint signaling, and stromal-myeloid exclusion within the tumor microenvironment. Taken together, the field is moving from empirical regimen selection toward a model that integrates disease tempo, patient fitness, translational biomarkers, and mechanism-based sequencing. Future progress will depend on composite biomarker validation, biomarker-enriched trials, rational resistance-directed combinations, and structural measures that improve external validity and equitable access.

## Introduction

Studying immunotherapy in renal cell carcinoma (RCC) has become a major priority because it offers durable benefit in a disease that historically responded poorly to conventional systemic therapy (1). Over the last two decades, management evolved from cytokine-based approaches to vascular endothelial growth factor (VEGF)-directed and mammalian target of rapamycin (mTOR)-directed therapies, and subsequently to immune checkpoint inhibitor (ICI)-based combinations (2, 3). Pivotal studies such as CheckMate-214, KEYNOTE-426, CheckMate-9ER, and CLEAR established the contemporary first-line treatment paradigm in metastatic RCC (mRCC) (4–6).

Clear cell RCC (ccRCC) is characterized by a highly immunogenic tumor microenvironment (TME), which provides the biological rationale for immunotherapeutic strategies (7, 8). Nevertheless, metastatic disease remains lethal for many patients, and durable remission is still limited to a subset of treated individuals because primary and acquired resistance remain common (9, 10).

We conducted a literature-based narrative review using PubMed, review articles, and major clinical trial reports focused on immunotherapy in RCC. The synthesis integrates pivotal clinical trials, translational biomarker studies, resistance biology, and real-world evidence to assess both efficacy and clinical applicability.

A central challenge is not simply whether ICI-based therapy works, but which regimen is most appropriate for which patient, under which clinical circumstances, and with what expectation of real-world effectiveness. In this revised version, greater emphasis is placed on cross-trial heterogeneity, patient selection, external validity, biomarker interpretability, and the translational relevance of resistance mechanisms, because these issues determine whether trial results can be converted into routine clinical benefit.

## Clinical efficacy of first-line immunotherapy combinations

We have strong phase III evidence supporting current first-line ICI-based strategies. CheckMate-214 showed that nivolumab plus ipilimumab (Nivo+Ipi) produced substantial long-term survival benefit, particularly in intermediate/poor-risk disease, with a hazard ratio (HR) for overall survival (OS) of 0.71 in the intent-to-treat population and 0.69 in the intermediate/poor-risk subgroup (11). KEYNOTE-426 demonstrated that pembrolizumab plus axitinib significantly improved OS and progression-free survival (PFS) compared with sunitinib, with an OS HR of 0.73 (12). CheckMate-9ER and CLEAR further confirmed the efficacy of ICI-TKI combinations, each reporting an OS HR of 0.66 versus sunitinib (12).

These trials collectively established ICI-based combinations as the therapeutic backbone of first-line mRCC and showed that regimen class matters clinically: dual ICI is associated with durable long-term remission in selected patients, whereas ICI-TKI combinations deliver higher initial response rates and faster tumor shrinkage across broader risk categories (13, 14).

Direct comparison of ICI-ICI and ICI-TKI regimens requires caution because there are no head-to-head randomized trials and because the major studies differ in IMDC risk distribution, follow-up duration, endpoint maturity, subsequent therapies, and the proportion of patients with favorable-risk disease. CheckMate-214 generated its most practice-defining signal in intermediate/poor-risk disease, whereas KEYNOTE-426, CheckMate-9ER, and CLEAR enrolled all IMDC groups and are often selected in practice when broad activity and rapid cytoreduction are prioritized. Accordingly, apparent differences in response rate or survival curves should be interpreted as hypothesis-generating rather than as proof of universal superiority of one class over another (11*;*^12^*;*^15^*).*

Toxicity profiles also differ in ways that are clinically decisive. Dual ICI concentrates immune-related adverse events early and may require corticosteroid-intensive management, whereas ICI-TKI regimens introduce the ongoing burden of TKI-associated hypertension, diarrhea, fatigue, hepatotoxicity, mucositis, and dose modification. In practical terms, treatment choice should therefore integrate disease tempo, symptom burden, IMDC risk, autoimmune history, cardiovascular comorbidity, and the feasibility of prolonged monitoring in routine care rather than rely on efficacy endpoints alone, **Table 1** (16, 17).

**Table 1:** Critical comparison of pivotal first-line phase III regimens in mRCC.

Trial	Regimen class / control	IMDC context and cross-trial caveat	Key efficacy signal	Toxicity / patient-selection note	Reference
CheckMate-214	ICI-ICI (nivolumab + ipilimumab) vs sunitinib	All risk groups; clinically defining benefit most robust in IMDC intermediate/poor risk	OS HR 0.69 (int/poor); 0.71 (ITT)	Durable remission potential; lower early ORR than some ICI-TKI regimens; immune-related toxicities can be front-loaded	11
KEYNOTE-426	ICI-TKI (pembrolizumab + axitinib) vs sunitinib	All IMDC groups; cross-trial comparison limited by broader-risk enrollment	OS HR 0.73	High ORR and rapid tumor shrinkage; chronic TKI toxicity and dose-modification burden matter in long-term care	12
CheckMate-9ER	ICI-TKI (nivolumab + cabozantinib) vs sunitinib	All IMDC groups; no direct randomized comparison with ICI-ICI regimens	OS HR 0.66	Broad activity plus VEGF/MET/AXL blockade; useful when early disease control is important, but long-term TKI tolerability is relevant	12
CLEAR	ICI-TKI (pembrolizumab + lenvatinib) vs sunitinib	All IMDC groups; highly active regimen but cross-trial ranking remains indirect	OS HR 0.66	Marked tumor shrinkage; efficacy must be balanced against cumulative toxicity and dose intensity considerations	12

**Figure 1:**
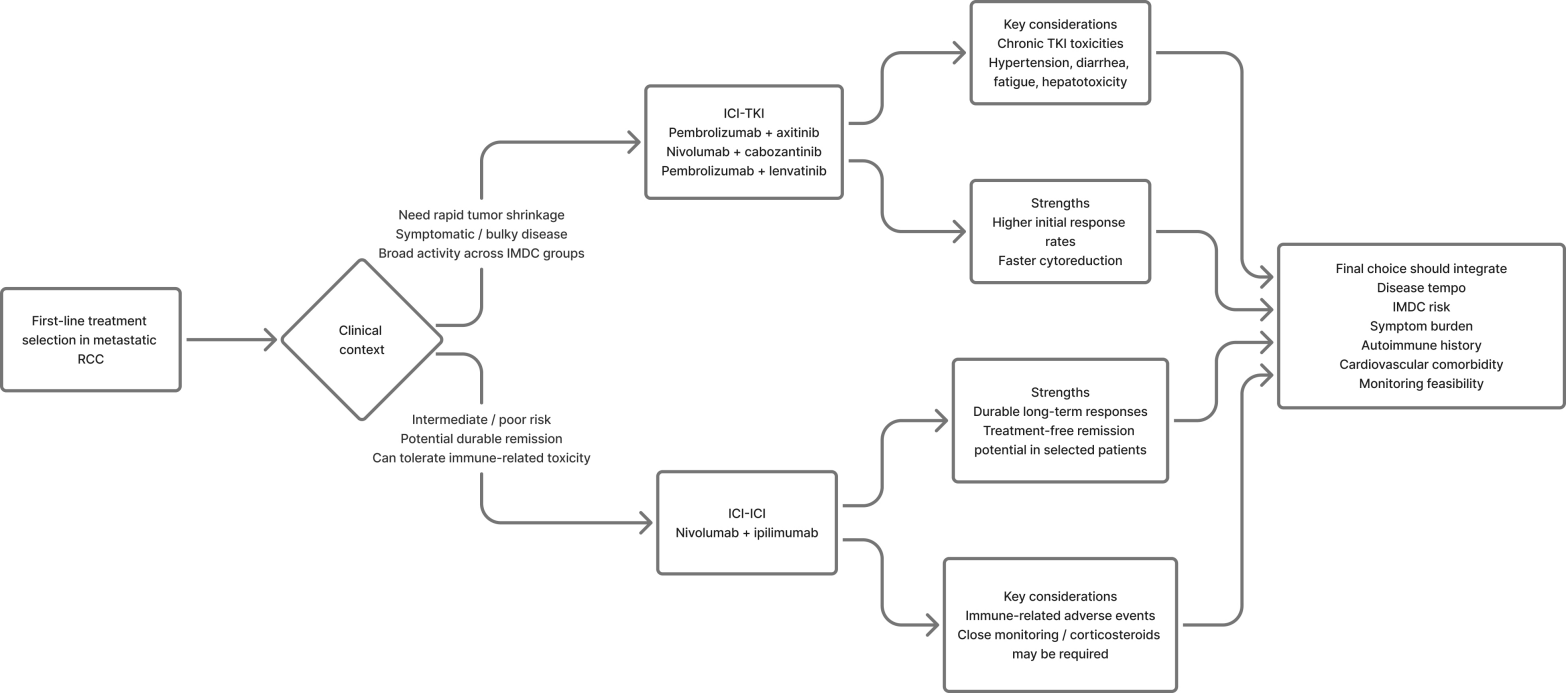
First-line treatment algorithm in metastatic renal cell carcinoma. This figure summarizes the practical clinical logic for selecting between dual immune checkpoint blockade (ICI-ICI) and immune checkpoint inhibitor–;tyrosine kinase inhibitor combinations (ICI-TKI) in the first-line setting. ICI-ICI is generally favored when durable long-term remission is prioritized, particularly in intermediate- or poor-risk disease, whereas ICI-TKI is often preferred when rapid tumor shrinkage and broader activity across IMDC risk groups are required. Final treatment choice should integrate disease tempo, symptom burden, IMDC risk, autoimmune history, cardiovascular comorbidity, and feasibility of monitoring.

Study-specific interpretation further sharpens this distinction. In CheckMate-214, the clearest signal for durable benefit emerged in IMDC intermediate/poor-risk disease, where nivolumab plus ipilimumab generated the most practice-defining long-term survival and complete-response data, making it especially relevant when durable remission potential is prioritized. By contrast, KEYNOTE-426, CheckMate-9ER, and CLEAR more consistently support the clinical logic of ICI-TKI when rapid shrinkage, broader all-comer activity, or early disease control is needed. Favorable-risk disease remains more nuanced: pooled and trial-level analyses suggest that early disease-control advantages of VEGF-containing regimens do not necessarily translate into the same long-tail remission logic seen with dual ICI, but cross-study duration-of-response comparisons remain unstable because follow-up maturity, censoring, and subsequent therapy differ across trials. This is why regimen selection should be anchored to the specific trial context being cited rather than generalized across all IMDC groups (11, 12, 18). This practical selection logic is summarized in **Figure 1**.

## Real-world evidence and the efficacy-effectiveness gap

Real-world evidence (RWE) distinguishes efficacy in controlled trials from effectiveness in routine practice. Registration studies often exclude patients with poor performance status, substantial comorbidity burden, untreated brain metastases, or other factors that complicate therapy delivery, whereas RWE captures these clinically relevant populations (19, 20).

Comparative real-world analyses suggest that ICI-TKI regimens may demonstrate higher objective response rates, partly because physicians preferentially use them in patients who need rapid disease control; however, in intermediate- and poor-risk subgroups, consistent overall survival superiority over dual ICI has not been established (21, 22).

**Figure 2:**
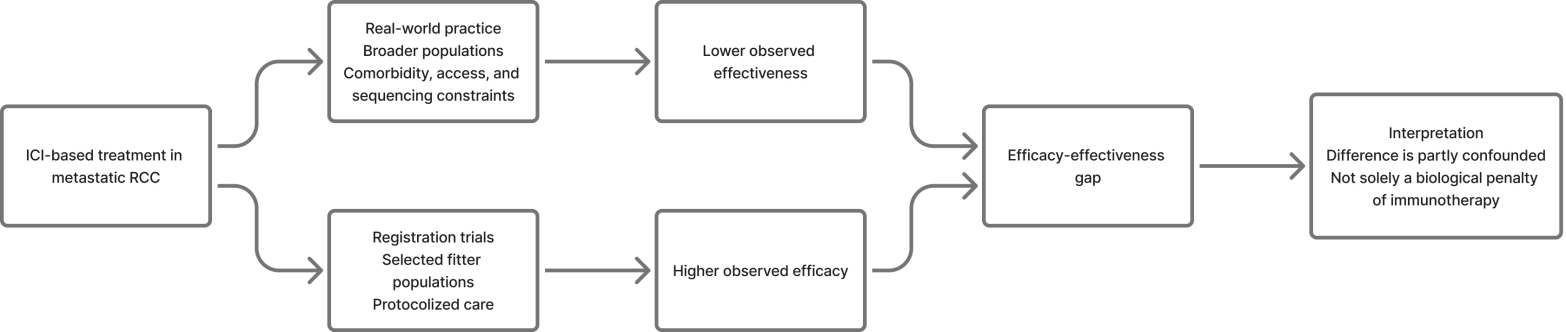
Efficacy versus real-world effectiveness in metastatic renal cell carcinoma.This figure summarizes the conceptual difference between efficacy observed in registration trials and effectiveness observed in routine clinical practice. Registration trials typically reflect outcomes in fitter, selected populations treated under protocolized conditions, whereas real-world practice includes broader populations affected by comorbidity burden, access barriers, and sequencing variability. The figure emphasizes that the efficacy-effectiveness gap is partly confounded and should not be interpreted as solely a biological penalty of immunotherapy.

The frequently cited observation that overall survival in routine care may be roughly 30-40% lower than trial-based estimates should not be interpreted as a universal biological penalty of immunotherapy. Rather, it is partly explained by case-mix and health-system confounding: poorer ECOG performance status, higher comorbidity burden, delayed referral, access barriers, underrepresentation of vulnerable groups in registrational studies, treatment interruption from toxicity, and non-uniform sequencing after first-line failure. The most balanced interpretation is that trial efficacy approximates the upper boundary of achievable benefit, whereas real-world effectiveness reflects the combined influence of tumor biology, patient fitness, and the health system’s ability to deliver complex care (19–21, 23). This efficacy-effectiveness framework is summarized in **Figure 2**.

## Efficacy in non-clear cell RCC histologies

Non-clear cell RCC (nccRCC) comprises a heterogeneous group of biologically distinct subtypes, including papillary, chromophobe, and translocation RCC, and collectively accounts for approximately 20-25% of kidney cancers (24, 25). Because these tumors were historically underrepresented or excluded from pivotal studies, major evidence gaps persist.

Earlier immunotherapy studies in nccRCC showed modest activity. In KEYNOTE-427 cohort B, pembrolizumab monotherapy achieved an objective response rate (ORR) of 26.7%, while CheckMate-920 reported an ORR of 19.6% for nivolumab plus ipilimumab in mixed nccRCC populations (26). Chromophobe RCC in particular behaved as an immune-cold subtype with relatively limited benefit.

More recently, KEYNOTE-B61 provided an important proof of concept for ICI-TKI combinations in nccRCC, with pembrolizumab plus lenvatinib yielding an ORR of 50.6% and a median PFS of 17.9 months. Activity was especially encouraging in papillary and translocation RCC, and even chromophobe RCC showed a therapeutic signal, **Table 2** (27, 28).

**Table 2:** Immunotherapy efficacy in non-clear cell RCC.

Trial / study	Regimen	Histology subtype	ORR	Key finding	Reference
KEYNOTE-427 (Cohort B)	Pembrolizumab monotherapy	Mixed nccRCC	26.7%	Modest activity; highlighted need for combinations	26
CheckMate-920	Nivolumab + ipilimumab	Mixed nccRCC	19.6%	Lower efficacy than typically seen in clear cell RCC	26
KEYNOTE-B61	Pembrolizumab + lenvatinib	Mixed nccRCC	50.6%	Important efficacy signal for ICI-TKI in a previously underserved population	27
KEYNOTE-B61 subgroup	Pembrolizumab + lenvatinib	Papillary RCC	~54%	Particularly encouraging activity in the most common nccRCC subtype	28
KEYNOTE-B61 subgroup	Pembrolizumab + lenvatinib	Chromophobe RCC	Signal detected	Suggests that combination therapy may partially overcome immune-cold biology	27

## Biomarkers for immunotherapy in RCC

RCC remains unusual among immunotherapy-sensitive malignancies because no single validated predictive biomarker is used routinely to select patients for ICI therapy. Unlike melanoma or lung cancer, markers such as PD-L1 expression and tumor mutational burden (TMB) have shown inconsistent and clinically insufficient performance in RCC (29, 30).

Viewed comparatively, current RCC biomarker candidates fall into three evidence tiers. PD-L1 expression and TMB occupy the lowest tier for clinical decision-making because, despite biological plausibility, their predictive signals are inconsistent across studies, assay platforms, and cut points, and neither marker reproducibly identifies a population that should receive or avoid a specific ICI regimen. A second tier includes PBRM1 alterations, VHL-related angiogenic or hypoxia programs, and gene-expression or spatial immune signatures: these are biologically informative and repeatedly hypothesis-generating, but most supporting data remain retrospective, platform-dependent, and insufficiently standardized for prospective use. The most conceptually attractive tier is therefore the composite biomarker approach, in which genomic, transcriptomic, and spatial features are integrated to reflect the multidimensional biology of RCC; however, this approach is still exploratory because prospective validation and deployment-ready assays are lacking. Framed this way, the core problem is not the absence of candidate biomarkers, but the absence of reproducible and clinically transportable biomarker systems (31*;*^32^*;*^33^*).*

The so-called RCC biomarker paradox reflects the observation that many patients benefit from immunotherapy despite relatively low TMB, implying that immunogenicity in RCC is driven by factors not captured by mutation burden alone.

PD-L1 expression has some biological and prognostic relevance, but assay variability, different cut points, intratumoral heterogeneity, and continued responses in PD-L1-negative tumors prevent its use as a reliable treatment-selection biomarker. TMB is even less useful in RCC because mutation burden does not capture pseudohypoxia-driven inflammation, endogenous retroelement biology, or the spatial organization of the immune microenvironment (31, 32).

PBRM1 illustrates why descriptive biology does not automatically translate into clinical utility. Loss-of-function alterations have been associated in some cohorts with improved response to immune-based therapy, plausibly through chromatin remodeling and interferon-related effects; however, the association is inconsistent across retrospective datasets, treatment backbones, and analytic methods. At present, PBRM1 is biologically informative but clinically exploratory rather than validated (31, 33). 

**Figure 3:**
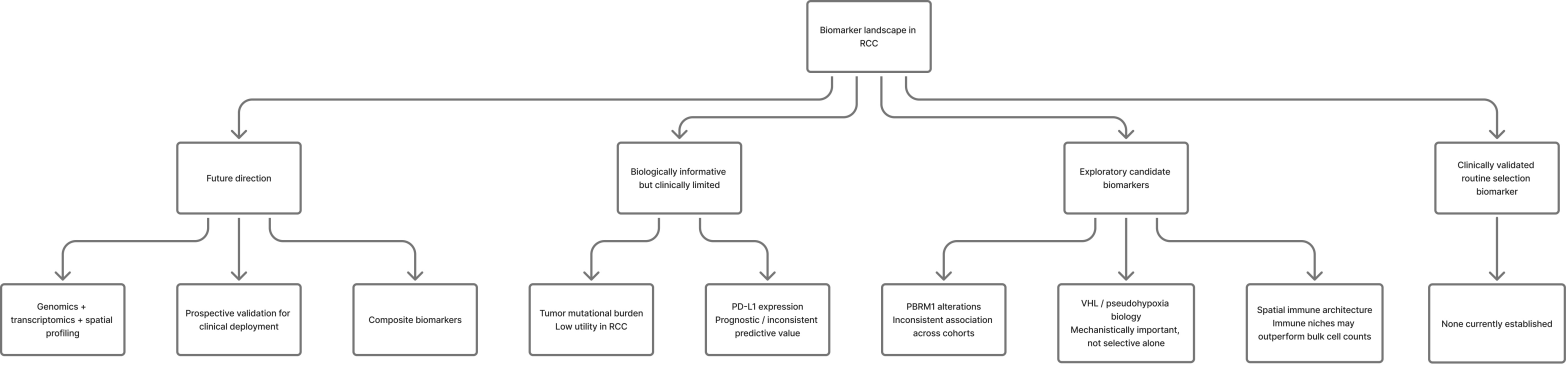
Biomarker landscape in renal cell carcinoma. This figure summarizes the current biomarker framework in renal cell carcinoma. No single biomarker is currently validated for routine clinical selection of immune checkpoint inhibitor therapy. PD-L1 expression and tumor mutational burden have biological or prognostic relevance but limited predictive clinical utility, whereas PBRM1 alterations, VHL-driven pseudohypoxia, and spatial immune architecture remain exploratory but mechanistically informative candidates. The figure also highlights the likely future direction of the field toward composite biomarkers integrating genomics, transcriptomics, and spatial profiling with prospective clinical validation.

VHL loss is central to clear cell RCC biology but has limited value as a stand-alone predictive biomarker because it is so common. Its importance is mechanistic: it explains pseudohypoxia, angiogenesis, and metabolic rewiring that may shape sensitivity to VEGF-directed therapy, HIF-2 inhibition, and immune combinations. Similarly, spatial immune architecture may be more informative than bulk CD8+ cell density, because response appears to depend on organized immune niches rather than on cell counts alone. These observations support a structured move toward composite biomarkers that integrate genomics, transcriptomics, and spatial profiling rather than continued reliance on single-marker paradigms (29, 32, 33). A structured summary of candidate biomarkers, their rationale, level of evidence, and current clinical applicability is provided in **Table 3**, and a schematic overview of the current biomarker landscape is presented in **Figure 3**.

**Table 3:** Resistance mechanisms with explicit clinical implications.

Resistance setting	Mechanism	Biological basis	Clinical / therapeutic implication	Key references
Primary	Immune-cold / excluded phenotype	Poor effector T-cell infiltration; stromal restriction	Favor TME-modulating combinations or trial enrollment rather than repeated ICI-only approaches	34, 35
Primary	Antigen-presentation deficit	MHC-related impairment; interferon insensitivity	Low likelihood of benefit from immune activation alone; consider mechanism-switch strategies	36
Primary	Suppressive cytokine program	High IL-10 / TGF-beta; myeloid and stromal suppression	Rationale for stromal or cytokine-directed combinations in biomarker-enriched trials	47
Acquired	Immunoediting / B2M-MHC-I loss	Tumor escape after initial immune pressure	Supports study of NK-cell or other MHC-independent strategies; local therapy may be useful for oligoprogression	37
Acquired	Compensatory checkpoints	LAG-3, TIGIT, TIM-3 upregulation after PD-1 blockade	Rationale for next-generation checkpoint inhibitor trials after frontline ICI exposure	29
Tumor-intrinsic	VHL-HIF pseudohypoxia / Warburg metabolism	Angiogenic and adenosine-rich metabolic rewiring	Supports VEGF/HIF-2 or metabolic targeting as rational sequencing partners	38, 39
TME-mediated	CAF / TGF-beta / myeloid barrier	Physical and chemical exclusion of antitumor immunity	Supports stromal- and myeloid-directed combinations and biomarker-stratified trial design	35, 40

## Immunotherapy resistance: primary vs. acquired

Primary resistance describes tumors that fail to respond from treatment initiation, whereas acquired resistance develops after an initial period of response or disease control. In RCC, primary resistance may arise from immune exclusion, impaired antigen presentation, defective interferon signaling, suppressive cytokine states, or stromal barriers that prevent productive T-cell infiltration (34, 36).

Acquired resistance reflects evolutionary escape under therapeutic pressure and may involve immunoediting, loss of beta-2 microglobulin (B2M), reduced MHC-I expression, compensatory checkpoint upregulation, or broader phenotypic and metabolic adaptation (29, 37).

This distinction is clinically important because it should influence sequencing strategy. Primary resistance argues against repeated recycling of similar immune-only approaches and supports a mechanism switch toward VEGF/HIF-directed therapy, trial enrollment, or combinations designed to remodel the microenvironment. Acquired resistance after an initial durable response may justify local therapy for oligoprogression, continuation of effective systemic control for non-progressing disease compartments, or biologically selected trials targeting compensatory checkpoints such as LAG-3 or TIGIT (15, 29, 41).

## Tumor-intrinsic resistance mechanisms

Clear cell RCC is fundamentally shaped by VHL inactivation and constitutive hypoxia-inducible factor signaling, which produce pseudohypoxia, VEGF upregulation, and metabolic reprogramming. This biology promotes aerobic glycolysis, altered lipid handling, glutamine dependence, and accumulation of immunosuppressive metabolites such as lactate and adenosine, thereby impairing antitumor immunity (38, 39, 42).

Tumor-intrinsic escape may also occur through immunoediting and defective antigen presentation, including B2M-associated loss of MHC class I expression, which reduces CD8+ T-cell recognition.

The translational consequence is that metabolic and antigen-presentation defects should not be viewed as purely descriptive biology. They provide a rationale for mechanism-based combinations, including HIF-2 inhibition, adenosine pathway targeting, and MHC-independent strategies such as NK-cell-directed approaches. Future sequencing studies should prospectively test whether pseudohypoxic-angiogenic tumors derive differential benefit from VEGF- or HIF-2-directed salvage after frontline ICI exposure (39, 43).

## TME-mediated resistance mechanisms

In the RCC tumor microenvironment, cancer-associated fibroblasts (CAFs), transforming growth factor-beta (TGF-beta), VEGF-driven myeloid recruitment, and stromal remodeling can all contribute to a T-cell-excluded phenotype. Hypoxia-related VEGF and CCL2 signaling recruits myeloid-derived suppressor cells and M2-polarized macrophages, while dense extracellular matrix and CAF-mediated barrier formation prevent effective immune-cell penetration (35, 40, 44).

These resistance pathways should not be interpreted as isolated parallel lists, because in RCC they are biologically interconnected. VHL-driven pseudohypoxia and VEGF signaling can intensify myeloid recruitment and stromal remodeling, which then reinforce T-cell exclusion and create selective pressure for adaptive checkpoint compensation. From a clinical perspective, the angiogenic-myeloid-exclusion axis is currently the most actionable resistance program because it helps explain the benefit of VEGF-containing combinations and supports TME-directed salvage strategies after PD-1-based therapy. Alternative checkpoint upregulation is the next most actionable mechanism in the post-ICI setting, whereas deep antigen-presentation loss or entrenched tumor-intrinsic immune escape may be biologically profound but are less readily reversible in routine practice and therefore more likely to justify mechanism switching or trial enrollment than simple intensification of existing immunotherapy. This comparative framework moves resistance biology from a catalog of mechanisms toward a prioritization model for sequencing and rational combination design (45, 29, 46, 47).

Adaptive resistance after PD-1 blockade may further emerge through upregulation of alternative checkpoints such as LAG-3, TIGIT, and TIM-3, which sustain T-cell exhaustion despite ongoing PD-1 inhibition (29, 48).

**Figure 4:**
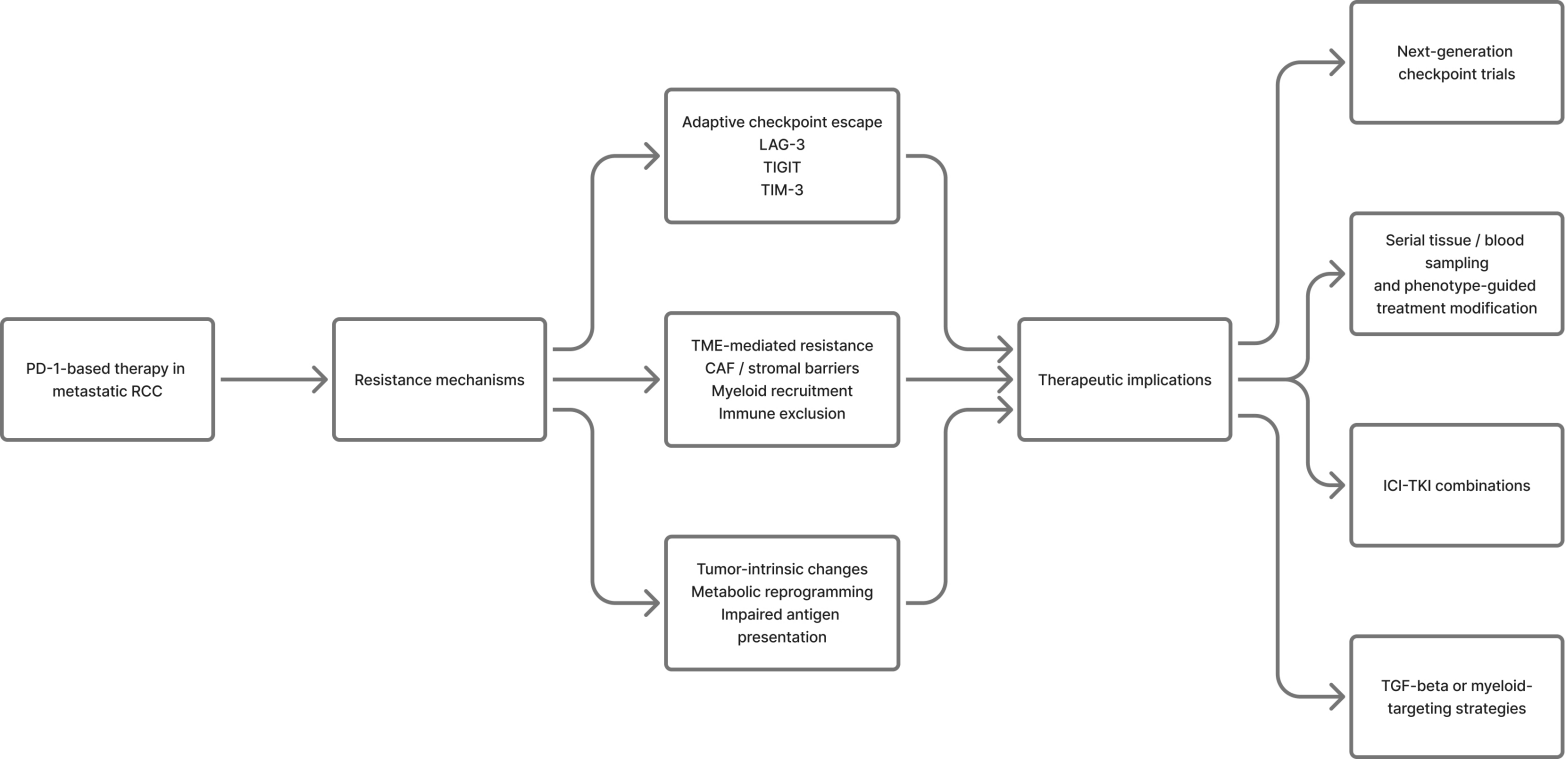
Resistance mechanisms and therapeutic implications in metastatic renal cell carcinoma. This figure summarizes major resistance pathways to PD-1-based therapy in renal cell carcinoma, including tumor-intrinsic changes, tumor microenvironment-mediated immune exclusion, and adaptive upregulation of alternative checkpoints such as LAG-3, TIGIT, and TIM-3. It also highlights the corresponding clinical implications, including the rationale for ICI-TKI combinations, TGF-beta- or myeloid-targeting strategies, next-generation checkpoint trials, and phenotype-guided treatment modification supported by serial tissue or blood sampling.

These TME-mediated mechanisms have immediate clinical implications. They support the biological logic of ICI-TKI combinations, justify investigation of TGF-beta or myeloid-targeting strategies in immune-excluded tumors, and argue for next-generation checkpoint trials in patients whose disease progresses after PD-1-based therapy. They also imply that future studies should stratify patients prospectively by inflamed versus excluded immune phenotypes and incorporate serial tissue or blood sampling to link resistance states to rational treatment modification (45, 49, 50). A concise translational summary of resistance mechanisms and their therapeutic implications is provided in **Table 4**, and an integrated schematic overview of resistance pathways and treatment implications is presented in **Figure 4**.

**Table 4:** Practical decision matrix for first-line ICI-ICI versus ICI-TKI selection in mRCC.

Decision domain	Considerations favoring ICI-ICI	Considerations favoring ICI-TKI	Interpretive caveat	Key sources
Need for rapid tumor shrinkage	Less compelling when disease tempo is indolent	More compelling when symptomatic burden or organ-threat requires early cytoreduction	Cross-trial ORR differences do not equal direct superiority	12, 21
Durable treatment-free control	Potential long-term remission / TFS is a major attraction	Chronic TKI exposure may limit TFS even when disease is controlled	Best candidates remain imperfectly defined	11, 15
Comorbidity and monitoring burden	May be acceptable when chronic TKI toxicity is undesirable	May be preferable when autoimmune risk or need for prompt control outweighs TKI burden	Both classes require careful toxicity infrastructure	16; 17
IMDC risk context	Evidence base is especially strong in intermediate / poor risk	Broad all-risk activity across pivotal trials	IMDC alone is insufficient for full patient selection	11, 12
External validity	Trial-like, closely monitored patients may capture best-case benefit	Real-world use may be shaped by access, dose modification, and monitoring feasibility	Routine-care outcomes are influenced by confounding beyond regimen biology	19, 20

## Unresolved clinical questions and decision points

Rather than reiterating prior summary points, the current field can be organized around a small number of unresolved clinical questions. First, which patients derive the greatest long-term advantage from ICI-ICI versus ICI-TKI? Available evidence suggests a trade-off between durable treatment-free remission potential and rapid cytoreduction, but no randomized head-to-head trial has resolved this decisively. Second, how should therapy be sequenced after failure of a frontline ICI-containing regimen? Third, which biomarker platform has the greatest chance of clinical deployment: limited genomic markers, gene-expression programs, spatial immune profiling, or a composite multi-omic assay? Fourth, how can the benefits seen in pivotal trials be extended to older, frailer, or socioeconomically disadvantaged patients who are underrepresented in registration studies? Framing the controversy in this way improves coherence and keeps attention on clinically unresolved problems rather than on repetitive restatement of established concepts (15, 21, 33). A practical treatment-selection framework is provided in **Table 5**.

**Table 5:** Structured summary of candidate biomarkers in RCC immunotherapy.

Biomarker	Biological rationale	Evidence synthesis	Clinical applicability	Representative references
PD-L1 expression	Reflects adaptive immune engagement and checkpoint biology	Associations with outcome are inconsistent across assays and studies; responses occur in PD-L1-negative disease	Exploratory; not validated for routine treatment selection	31, 32
Tumor mutational burden (TMB)	Surrogate for neoantigen load in some cancers	Generally low in RCC and poorly correlated with ICI benefit	Not clinically useful as a stand-alone biomarker in RCC	31
PBRM1 alterations	Chromatin remodeling, interferon response, immune modulation	Biologically plausible but effect size and direction vary across retrospective cohorts	Exploratory / hypothesis-generating	31, 33
VHL / HIF axis	Defines pseudohypoxia, angiogenesis, and metabolic rewiring in ccRCC	Mechanistically central but insufficient as a stand-alone predictor of ICI response	Mechanistic relevance; indirect therapeutic relevance rather than validated predictive use	31, 33
Gene-expression / immune signatures	Capture inflamed, angiogenic, or myeloid-rich tumor states	Promising but not yet standardized across platforms or trials	Exploratory; potential component of future composite assays	29, 32
Spatial immune architecture	Assesses functional immune niches and cell-cell proximity	May outperform bulk immune-cell counts in translational analyses	Exploratory but clinically compelling for future assay development	29, 32

## Theoretical and practical implications

Clinical success with VEGF-ICI combinations reinforces the concept that TME modulation is not ancillary but central to effective therapy in RCC. VEGF inhibition may normalize vasculature, reduce immunosuppressive myeloid recruitment, and create a more permissive environment for T-cell activity (45, 51).

This conceptual model extends beyond current regimens and supports exploration of HIF-2-directed combinations, metabolic modulators, and microenvironment-targeting strategies as future partners for immunotherapy.

At the bedside, these concepts translate into a more disciplined selection framework: disease tempo, symptom burden, autoimmune history, organ reserve, access to specialist toxicity management, and anticipated sequencing options should all be incorporated before choosing a first-line regimen. In parallel, trial design should move from empiric agent stacking toward biomarker-informed combination logic.

## Socioeconomic landscape: cost, access, and disparities

ICI-based combination regimens impose substantial financial toxicity. Health-economic analyses suggest that many of these strategies do not meet conventional cost-effectiveness thresholds against sunitinib unless substantial price reductions occur (52–54).

Logistical barriers also matter. Infusion-based treatment schedules, specialist follow-up, laboratory monitoring, and toxicity management disproportionately burden patients with limited transportation, reduced social support, or residence far from tertiary centers (55, 56).

Systemic inequities in referral, insurance approval, and representation in clinical trials further widen the gap between scientific progress and population-level benefit.

## Limitations and future directions

Key limitations in the field include the absence of head-to-head randomized comparisons among first-line combination regimens, the heavy reliance on retrospective biomarker datasets, and limited evidence on optimal sequencing after failure of frontline ICI-containing therapy.

Future work should prioritize biomarker-enriched prospective trials, standardized and clinically deployable composite biomarker platforms, longitudinal paired-biopsy studies, dedicated trials for non-clear cell subtypes, and endpoints such as treatment-free survival that better capture durable patient-centered benefit.

Equally important, future studies must improve external validity by deliberately enrolling older adults, patients with poorer performance status, and socioeconomically underrepresented populations. Without that shift, the efficacy-effectiveness gap will remain partly structural even if biologic resistance is better understood. The next step is to convert these biologic and clinical signals into a hierarchy of actionability that distinguishes broadly useful observations from biomarkers or resistance mechanisms that can truly guide sequencing and combination choice.

## Conclusion

First-line immunotherapy has transformed the treatment of metastatic RCC, but the central clinical problem has moved from proving that ICI-based therapy works to determining which regimen is best for a given patient and how resistance should be anticipated and managed.

Progress now depends on integrating a critical comparison of ICI-ICI and ICI-TKI regimens, a more nuanced interpretation of real-world effectiveness, structured biomarker development, and translationally informed sequencing strategies.
